# Utilization of Renal Replacement Therapy and Its Impact on the Emergency Department Length of Stay in South Korean Emergency Medical Centers

**DOI:** 10.3390/medicina62071273

**Published:** 2026-07-01

**Authors:** Ji Eun Kim, Jinwoo Jeong, Yuri Choi, Hyung Jun Moon

**Affiliations:** 1 Department of Emergency Medicine, Dong-A University Hospital, Twenty-Six Daesingongwon-Ro, Seo-Gu, Busan 49201, Republic of Korea; jinwoo@dau.ac.kr (J.J.); yurichoi@dau.ac.kr (Y.C.); 2 Department of Emergency Medicine, Ajou University Hospital, Suwon 16499, Republic of Korea; mhj@ajou.ac.kr

**Keywords:** renal replacement therapy, hemodialysis, continuous renal replacement therapy, emergency department length of stay, emergency medical centers, acute kidney injury, emergency medicine, critical care, emergency department overcrowding, healthcare policy

## Abstract

*Background*: The utilization of renal replacement therapy (RRT) is crucial for the management of patients with acute kidney injury (AKI) in emergency departments (EDs). The prompt initiation of RRT, encompassing both intermittent hemodialysis (HD) and continuous renal replacement therapy (CRRT), is acknowledged as beneficial for critically ill patients. The purpose of this study is to investigate the implementation of RRT within EDs and its impact on ED length of stay (EDLOS) in South Korea. *Methods*: This retrospective study utilized data from the National Emergency Department Information System (NEDIS) for the year 2019 to assess the utilization of RRT in emergency medical centers (EMCs) across South Korea. The analysis focused on RRT, which includes intermittent HD and CRRT, as identified through insurance billing codes for patients treated during ED visits and subsequent admissions. EMCs were categorized into three groups based on the frequency of RRT sessions, and the median EDLOS was evaluated. *Results*: Among 5,937,569 ED visits to Level I and II emergency medical centers (EMCs), 40,130 cases (0.68%) received RRT. Of the 162 EMCs, 58 centers (35.8%) did not perform intermittent HD in the ED and 106 centers (65.4%) did not perform CRRT in the ED during the study period. Centers that frequently performed CRRT in the ED showed significantly longer EDLOS compared with centers that seldom or never performed CRRT (588 min [IQR 286–767] vs. 270 min [IQR 147–337] and 205 min [IQR 149–363], respectively; *p* = 0.01). Regional disparities in the availability of ED-based RRT were also observed across South Korea. *Conclusions*: The frequency of RRT administration in EMCs in South Korea varied by region and facility. ED-based RRT utilization was associated with longer EDLOS, particularly in centers frequently performing CRRT. These findings suggest that patient acuity, institutional characteristics, and RRT-related resource utilization should be considered when evaluating EMC performance based on EDLOS.

## 1. Introduction

Acute kidney injury (AKI) is a common and serious complication among hospitalized and critically ill patients and is associated with increased mortality, prolonged hospitalization, and greater healthcare resource utilization [1,2]. In severe cases, AKI may lead to life-threatening complications, including refractory hyperkalemia, severe metabolic acidosis, fluid overload, and uremic complications. Renal replacement therapy (RRT) remains the cornerstone of supportive management for these conditions and is often required when conventional medical treatment fails [2,3]. RRT encompasses both intermittent hemodialysis (HD) and continuous renal replacement therapy (CRRT). HD is generally used in hemodynamically stable patients, whereas CRRT is preferred for critically ill patients with hemodynamic instability, septic shock, acute respiratory distress syndrome, or acute liver failure. Furthermore, the use of RRT has expanded beyond traditional renal indications and is increasingly applied in patients with multiorgan dysfunction, severe intoxication, and complex metabolic disturbances [4,5].

While the optimal timing of RRT initiation in patients with AKI remains controversial, there is broad consensus that RRT should be initiated promptly in patients with life-threatening complications such as refractory hyperkalemia, severe metabolic acidosis, fluid overload, or severe intoxication [3,5,6]. Delayed initiation of RRT in these situations may result in further clinical deterioration and increased morbidity. Therefore, when immediate RRT is required in patients presenting to the emergency department (ED), timely access to dialysis services is essential.

In parallel, emergency departments worldwide have experienced increasing overcrowding and prolonged boarding times, which have emerged as major patient safety and public health concerns [7]. Critically ill patients frequently remain in the ED for extended periods while awaiting intensive care unit admission, transfer to tertiary centers, or inpatient bed availability. As a result, therapies that were traditionally initiated after hospital admission are increasingly being delivered in the ED. RRT is one such resource-intensive intervention that requires specialized equipment, trained personnel, and continuous monitoring. Consequently, the capability of an ED to provide timely RRT has become an important component of modern emergency and critical care systems.

While numerous EDs globally provide RRT to critically ill patients, limited information is available regarding the availability and utilization of RRT in emergency care settings [8]. Most previous studies have focused on clinical outcomes, timing of RRT initiation, or specific patient populations, while relatively little is known about how frequently RRT is actually provided in EDs and how its utilization varies across institutions and geographic regions. Understanding the real-world availability of RRT is particularly important because critically ill patients requiring urgent dialysis may experience delays in treatment when appropriate resources are unavailable. In addition, variation in institutional RRT capacity may contribute to regional disparities in emergency care and influence operational outcomes such as emergency department length of stay (EDLOS). Furthermore, nationwide assessments of ED-based RRT utilization remain scarce, limiting our understanding of variations in RRT availability and their potential impact on emergency care delivery.

Therefore, this study examined national population-based data on ED visits in South Korea in 2019 to investigate the frequency and regional distribution of RRT utilization in emergency medical centers and to evaluate the association between RRT utilization and EDLOS.

## 2. Methods

### 2.1. Data Collection and Study Population

The National Emergency Department Information System (NEDIS) is a comprehensive database in South Korea that gathers and oversees real-time information from EDs across the country [9,10]. This retrospective study utilized data from NEDIS for the year 2019. EDs in South Korea are categorized into three groups based on hospital capabilities and functions, strategically located across provinces and metropolitan areas to provide essential emergency medical care nationwide. In 2019, South Korea had 402 EDs, including 38 Level I regional emergency medical centers, 124 Level II local emergency medical centers, and 240 Level III local emergency medical institutes [11]. The study included 162 emergency medical centers (EMCs), comprising 38 Level I and 124 Level II centers, while excluding smaller Level III local emergency medical institutes.

### 2.2. Statistical Variables

In 2019, we examined how often renal replacement therapy (RRT) was administered to patients visiting EMCs in South Korea, either during their ED stay or after hospital admission. RRT was divided into intermittent hemodialysis (HD) and continuous renal replacement therapy (CRRT), with CRRT including continuous HD and hemofiltration. We excluded hemoperfusion, which is used for specific therapeutic reasons (such as treating poisoning or removing cytokines), as well as peritoneal dialysis, continuous ambulatory peritoneal dialysis, and extracorporeal ascites dialysis. To evaluate the use of RRT, we analyzed the insurance billing codes for HD and CRRT reported to NEDIS, differentiating between RRT administered in the ED and those performed post-admission. Because some patients received RRT both during their ED stay and after hospital admission, these categories were not mutually exclusive.

We analyzed HD and CRRT utilization within EMCs. Multiple dialysis sessions performed for a single patient during the same hospital encounter were counted as a single case. The frequency of RRT utilization at each center was divided into three categories: “Never” for centers with no RRT cases in the year, “Seldom” for those with fewer than 12 cases, and “Frequent” for those with 12 or more cases. The threshold of 12 RRT cases per year was selected a priori as an operational definition to distinguish centers with regular RRT utilization from those with infrequent utilization, representing an annual RRT volume equivalent to approximately one case per month on average. Because no established criteria exist for categorizing center-level RRT utilization, this threshold was chosen to identify centers with at least minimal but recurring experience in providing RRT.

We examined the distribution of EMCs according to the frequency of RRT utilization. RRT use in EMCs was explored by classifying the country into 17 regions based on the administrative divisions of provinces and metropolitan cities.

The EMCs were categorized into three groups according to the frequency of RRT utilization, and the median EDLOS was evaluated. EDLOS was defined as the time interval from ED arrival to discharge from the ED after completion of treatment.

### 2.3. Statistical Analysis

Data processing and statistical analyses were performed using R version 4.2.1 (R Foundation for Statistical Computing, Vienna, Austria, 2022. Available at: https://www.R-project.org/, accessed on 1 July 2026).

Categorical variables are expressed as frequencies (%), while continuous variables are characterized by medians and interquartile ranges (IQR). The length of stay differences among the three ED groups were analyzed using the Kruskal–Wallis test, with subsequent post hoc analysis conducted via Dunn’s test. A significance level of 0.05 was established for both tests.

## 3. Results

### 3.1. General Characteristics

**Figure 1:**
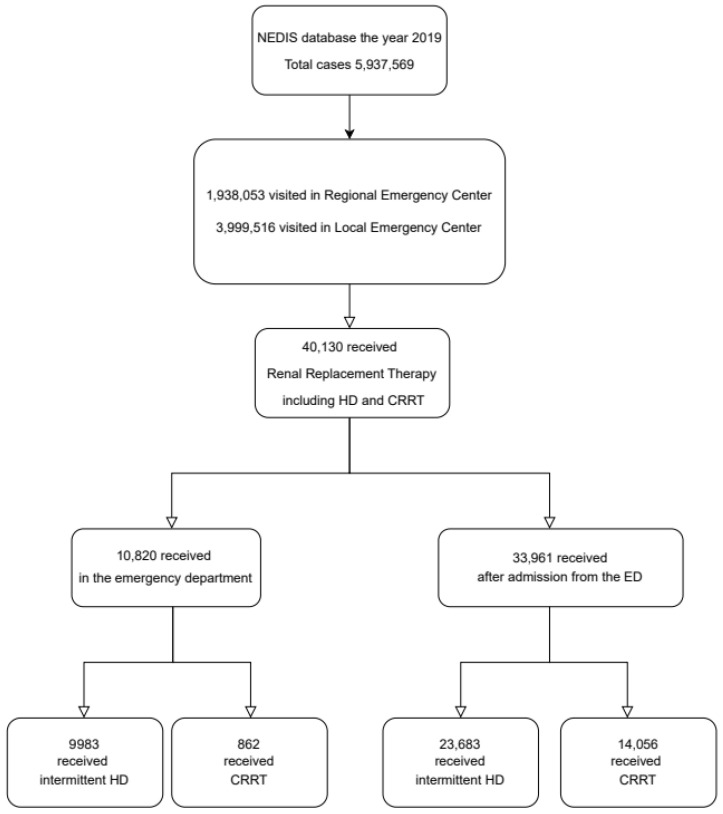
Flow diagram depicting the administration of renal replacement therapies (RRT) among study participants. Note that some patients received RRT in the ED and after admission. Additionally, it is important to highlight that some patients underwent both intermittent dialysis and continuous renal replacement therapy. Consequently, the total number of individual treatments may exceed the total number of cases. NEDIS—National Emergency Department Information System; HD—hemodialysis; CRRT—continuous renal replacement therapy; ED—emergency department.

In 2019, NEDIS reported 5,937,569 visits to Level I and Level II EMCs, with 40,130 cases (0.68%) receiving RRT. Of these, 10,820 cases received RRT in the ED, while 33,961 received RRT post-hospital admission (Figure 1). Because some patients received RRT both during their ED stay and after hospital admission, these categories were not mutually exclusive, and the summed counts exceed the total number of patients who received RRT. Table 1 summarizes the general characteristics of patients who received RRT in Level I and II EMCs.

### 3.2. Implementation of RRT by EMC Level

An analysis of RRT usage in EDs over a year (Table 2) reveals that out of 162 EMCs, 58 centers (35.8%) did not conduct any HD sessions, while 106 centers (65.4%) did not administer CRRT during patients’ ED stays. Notably, even among Level I centers, eight facilities (21.1%) did not provide HD, and 18 centers (47.4%) did not perform CRRT in the ED.

Conversely, using RRT after patient admission was more common (Table 3). Of the 162 centers, 130 (80.2%) and 115 (71.0%) conducted HD and CRRT at least 12 times over the year. This data suggests that hospitals tend to employ RRT more frequently post-admission, with CRRT showing notably greater variation in usage across centers. Among Level II centers, 79 (63.7%) implemented CRRT 12 times or more annually after admission, compared to 36 (94.7%) of Level I centers. It is also important to note that no Level I centers indicated a lack of CRRT post-admission.

The availability of CRRT in the ED was substantially more limited than that of intermittent HD. While 64.2% of EMCs provided HD in the ED during the study period, only 34.6% provided CRRT. This disparity was particularly evident among Level II EMCs, where 71.0% of centers did not perform any CRRT in the ED. These findings suggest that access to CRRT remains uneven across emergency care facilities.

### 3.3. Frequency of RRT in EMCs Across Administrative Regions in South Korea

**Figure 2:**
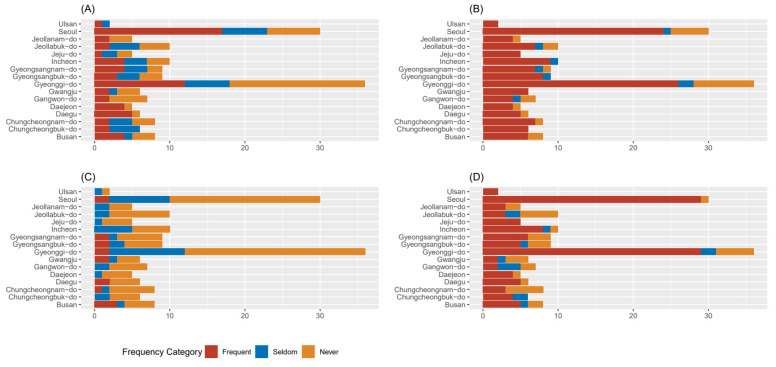
Regional Distribution of EDs: Utilization of RRT in South Korea (2019). This figure illustrates the frequency of renal replacement therapy (RRT) across various EMCs in South Korea during 2019. Each section below highlights the type and timing of RRT provided to patients: (**A**) Intermittent hemodialysis (HD) administered in the ED; (**B**) Intermittent HD following admission from the ED; (**C**) Continuous Renal Replacement Therapy (CRRT) in the ED; and (**D**) CRRT after admission from the ED.

The nation is segmented into regions comprising provinces and metropolitan cities, with EMCs in each segment classified into three categories—frequent, seldom, and never—according to the yearly utilization frequency of RRT. The findings illustrated in Figure 2 show that EMCs with frequent HD utilization existed across all regions. Importantly, in most areas—aside from Jeollanam-do and Gangwon-do—most centers conducted at least one HD session in the ED.

Conversely, the use of frequent CRRT in the ED was noted in fewer areas. Some regions lacked centers identified as frequent CRRT providers, and in many places, most centers did not offer any CRRT sessions in the ED. Moreover, the analysis shows that HD and CRRT were utilized more frequently following hospital admission than during ED visits, as seen in the distribution patterns across regions.

Regional variation was more pronounced for CRRT than for intermittent HD. Frequent HD utilization was observed across all administrative regions, indicating relatively broad access to conventional dialysis services throughout the country. In contrast, frequent CRRT utilization in the ED was concentrated in a limited number of regions, whereas several regions had no EMCs classified as frequent CRRT providers. These findings suggest substantial geographic variation in the availability of CRRT across emergency care facilities.

### 3.4. Analysis of Edlos Based on the Frequency of Rrt Utilization

**Figure 3:**
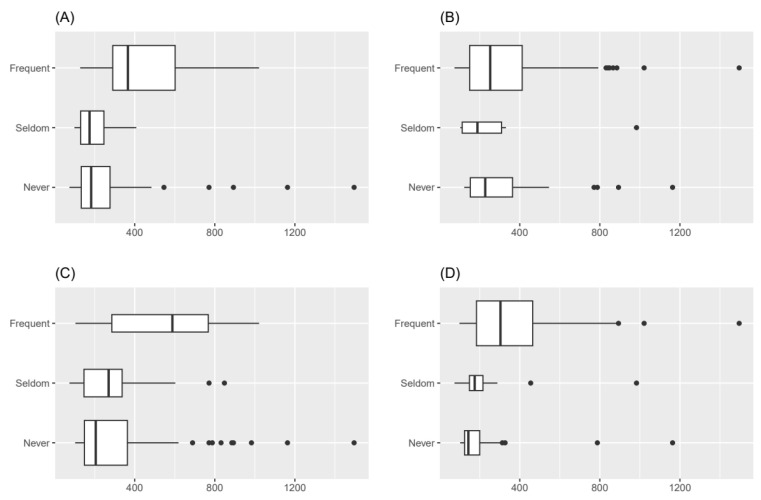
Relationship Between Renal Replacement Therapy Utilization and ED Length of Stay. This box plot illustrates the EDLOS categorized by the frequency of RRT. The vertical axis indicates the frequency of RRT, while the horizontal axis depicts the median EDLOS measured in minutes. (**A**) Intermittent Hemodialysis (HD) administered in the ED; (**B**) Intermittent Hemodialysis (HD) following admission from the ED; (**C**) Continuous Renal Replacement Therapy (CRRT) conducted in the ED; (**D**) Continuous Renal Replacement Therapy (CRRT) following admission from the ED.

Over one year, RRT utilization was classified into three categories: frequent, rare, and never. Boxplots represented the median EDLOS across all EMCs (Figure 3).

EMCs that frequently provide intermittent HD in the ED experienced an EDLOS roughly three hours longer than those that seldom or never offer IHDs. Specifically, the frequent group reported an EDLOS of 366 min [IQR 290—602], while the seldom group had an EDLOS of 174 min [IQR 130—246], and the never group had 182 min [IQR 133—277] (*p* < 0.001).

In contrast, when intermittent HD was initiated post-admission, the median length of stay showed no significant differences among the Frequent (252 min, IQR 150–412), Seldom (189 min, IQR 113–309), and Never groups (228 min, IQR 153–364) (*p* = 0.54).

Similarly, using CRRT in the ED led to a median length of stay (LOS) of 588 min [IQR 286–767] for the frequent group. This duration is over 5 h longer compared to the Seldom group (270 min, IQR 147–337) and the never group (205 min, IQR 149–363) with a *p*-value of 0.01. When CRRT was applied after admission, the frequent group’s median LOS decreased to 304 min [IQR 184–465], still more than 2 h longer than the Seldom group (175 min, IQR 149–216) and the never group (143 min, IQR 124–200), resulting in a *p*-value of <0.001).

Post hoc analyses showed no statistically significant differences between the seldom and never groups in any scenario. In contrast, the frequent group consistently had a significantly longer EDLOS compared to the other two groups, irrespective of whether treatment occurred in the ED or after admission.

## 4. Discussion

RRT is now utilized not only for patients with AKI but has recently expanded to support the heart, lungs, liver, and immune system as an adjunctive therapy [4]. Timing of RRT initiation in critically ill patients suffering from multiorgan failure can affect outcomes, yet definitive guidelines on the best initiation timing are still lacking [6,12]. Nevertheless, urgent HD is generally considered necessary in patients with life-threatening complications such as acute pulmonary edema due to fluid overload, severe hyperkalemia, and significant metabolic acidosis [13,14]. Emergency HD is also needed for acute poisoning and drug overdoses that do not respond to conventional treatment. Many studies have evaluated the potential benefits of early CRRT initiation, particularly in patients with hemodynamic instability [5,15,16]. Although previous studies have described the clinical role and indications of RRT in critically ill patients, national-level data regarding the actual utilization of ED-based RRT remain limited. Furthermore, direct comparisons between countries are challenging because the availability of ED-based RRT depends on institutional resources, staffing, critical care capacity, and local practice patterns.

RRT is resource-intensive and requires equipment, skilled personnel, physical space, and time. One major issue affecting patient care in EDs is overcrowding, which dramatically limits the ability to administer time-consuming RRT in these environments [17,18]. In this study, most EMCs administered HD and CRRT more often after patient admission than before. When there is a delay in patient admission due to various factors or when urgent RRT is needed, it must be included in the initial treatment in the ED. Consequently, EMCs that cannot provide RRT during a patient’s ED stay will likely encounter significant challenges in accepting and treating critically ill patients.

In South Korea, CRRT is primarily used for hemodynamically unstable patients and generally requires continuous monitoring and specialized staffing. Therefore, it is most commonly provided in emergency departments or intensive care units (ICUs), although institutional practices may vary. The study’s findings also indicated that the frequency of CRRT post-admission in EMCs significantly differs from that of HD. This implies that many hospitals might lack the necessary resources to provide CRRT in the ED. Consequently, hospitals without CRRT capabilities in the ED may face challenges in admitting patients who require CRRT when access to appropriate specialized care settings is limited. This limitation may lead to delays in both the transfer of patients to the ED and their subsequent treatment. Additionally, regional differences in CRRT availability suggest that some areas may face greater challenges in managing ED patients who require this treatment.

In addition to equipment availability, successful implementation of CRRT in the ED requires trained nephrologists, dialysis nurses, critical care physicians, and personnel capable of providing continuous monitoring. Therefore, the ability to provide CRRT may depend not only on physical resources but also on institutional staffing and organizational capacity. Variations in the availability of these resources may partially explain the substantial differences in CRRT utilization observed across EMCs and regions in this study.

Furthermore, in South Korea, the criteria for designating and evaluating EMCs focus solely on the availability of RRT equipment. They do not consider whether RRT is actually provided to ED patients. Therefore, the findings of this study suggest that current evaluation systems may not adequately reflect the actual delivery of RRT in the ED. Despite meeting equipment-based designation criteria, many centers either fail to provide RRT to ED patients or do so only to a very limited extent.

ED-based RRT utilization was associated with prolonged EDLOS. This study also demonstrated that facilities providing HD and CRRT in the ED showed significantly longer median EDLOS. Research has shown that larger hospitals and higher-level EDs often care for a greater proportion of critically ill patients, who also tend to experience prolonged EDLOS [11,19,20]. Furthermore, centers that frequently perform RRT, particularly CRRT, may function as higher-acuity referral centers caring for patients with greater illness severity and more complex clinical conditions requiring prolonged stabilization and intensive monitoring in the ED. Because hospital-level characteristics such as ICU capacity, ED volume, referral patterns, and patient case-mix were not available in the NEDIS database, these factors could not be incorporated into the analysis. Therefore, the observed association between frequent ED-based RRT utilization and prolonged EDLOS should be interpreted with caution, as it may reflect both the operational burden of RRT provision and underlying institutional characteristics.

In South Korea, a policy has been enacted to prevent overcrowding by penalizing hospitals with long EDLOS. Hospitals incur considerable financial losses if the mean EDLOS for admitted patients surpasses six hours, which may discourage investment in RRT capacity within the ED. To enhance access to RRT for critically ill patients in the ED, policy frameworks should recognize the resource-intensive nature of ED-based RRT and avoid creating disincentives associated with prolonged EDLOS for hospitals that provide such care.

Because patients requiring emergency RRT are often among the most critically ill patients treated in the ED, prolonged EDLOS in these cases may reflect the complexity of care rather than inefficient patient flow alone. Therefore, EDLOS-based performance evaluations should consider the clinical context and resource intensity of advanced organ support therapies such as RRT. Policy measures that recognize these cases as high-acuity, resource-intensive care may help prevent unintended disincentives for hospitals to provide timely ED-based RRT.

This study has the following limitations. Firstly, the examination of HD and CRRT use relied on insurance billing codes; however, the accuracy of these codes has not been validated in comparison with NEDIS, raising concerns about potential data entry errors in the NEDIS database. Nevertheless, since NEDIS data are validated against mandatory medical records during the assessment of EMCs, the risk of inaccuracies regarding whether dialysis was ultimately administered is considered low. Secondly, this study recognized EDLOS trends based on dialysis frequency at each hospital. Still, it did not consider various patient factors, hospital settings, or other treatments that could impact EDLOS. Consequently, establishing a causal relationship between these two factors is challenging. In addition, the threshold used to define frequent RRT utilization was based on an operational definition rather than an established standard. Alternative categorizations may have yielded different results, and categorization of a continuous utilization measure may have resulted in some loss of information. Furthermore, the study confirmed whether HD occurred while the patient was in the ED but did not specify if the dialysis treatments were administered in the ED or a dedicated dialysis unit. Due to limitations in the NEDIS data, the exact location where dialysis was performed could not be determined. Therefore, the findings do not ascertain the ED’s capability to deliver HD for patients in critical condition who need intensive monitoring in the ED, as opposed to the dialysis units.

## 5. Conclusions

In South Korea, the frequency of RRT administration in emergency medical centers varied across regions and facilities. Longer median EDLOS was observed in centers that frequently provided ED-based RRT; however, this association may reflect not only the operational burden of RRT provision but also differences in patient acuity, case complexity, referral patterns, and institutional characteristics. These contextual factors should be considered when interpreting EDLOS as a performance indicator, and policy efforts should support timely access to RRT for critically ill patients.

## Figures and Tables

**Table 1:** General Characteristics of Patients Receiving RRT in Level I and II EMCs in South Korea, 2019.

	Level I EMC (*n* = 18,845)	Level II EMC (*n* = 21,285)
Male	11,116 (58.99)	12,395 (58.23)
Age (years)		
0–19	106 (0.56)	57 (0.27)
20–44	1326 (7.04)	1272 (5.98)
45–64	6004 (31.86)	6783 (31.87)
65–74	4802 (25.48)	5451 (25.61)
over 75	6607 (35.06)	7722 (36.28)
Vehicle		
FDA ambulance	5219 (27.70)	6631 (31.15)
Other ambulance	5277 (28.00)	4491 (21.10)
Other non-ambulance	8349 (44.30)	10,163 (47.75)
ED disposition		
Discharge	1996 (10.59)	2523 (11.85)
Admission	16,612 (88.15)	18,573 (87.26)
General ward	9098 (54.77)	10,992 (59.18)
Intensive care unit	7474 (44.99)	7559 (40.70)
Another unit	40 (0.24)	22 (0.12)
Transfer to another hospital	142 (0.75)	101 (0.47)
Death	94 (0.50)	75 (0.35)
Unknown	1 (0.01)	13 (0.06)
RRT location		
Stay in the ED	5225 (27.73)	5595 (26.29)
After admission	16,157 (85.74)	17,804 (83.65)
RRT modality		
intermittent HD	4758 (25.25)	5225 (24.55)
CRRT	483 (2.56)	379 (1.78)
Median EDLOS (minutes)	388 [IQR: 232–684]	320 [IQR: 179–546]
Median Hospital LOS (days)	11.0 [IQR: 5.0–22.1]	10.8 [IQR: 4.9–22.2]

The values are represented as *n* (%). RRT—renal replacement therapy; EMC—emergency medical center; FDA—Food and Drug Administration; ED—emergency department; HD—hemodialysis; CRRT—continuous renal replacement therapy; EDLOS—emergency department length of stay. LOS—length of stay; IQR—interquartile range. Percentages for RRT location may exceed 100% because patients could receive RRT both in the ED and after hospital admission and were therefore included in more than one category.

**Table 2:** EMCs by RRT Frequency in the ED.

	Frequent (≥12 Cases/Year)	Seldom (<12 Cases/Year)	Never (No Case/Year)	Total
Intermittent HD				
Level I	25 (65.8)	5 (13.2)	8 (21.1)	38
Level II	42 (33.9)	32 (25.8)	50 (40.3)	124
Total	67 (41.4)	37 (22.8)	58 (35.8)	162
CRRT				
Level I	9 (23.7)	11 (28.9)	18 (47.4)	38
Level II	7 (5.6)	29 (23.4)	88 (71.0)	124
Total	16 (9.9)	40 (24.7)	106 (65.4)	162

The values are represented as *n* (%). EMC—emergency medical center; HD—hemodialysis; CRRT—continuous renal replacement therapy.

**Table 3:** Frequency of RRT in EMCs Following Admission.

	Frequent (≥12 Cases/Year)	Seldom (<12 Cases/Year)	Never (No Case/Year)	Total
Intermittent HD				
Level I	32 (84.2)	2 (5.3)	4 (10.5)	38
Level II	98 (79.0)	6 (4.8)	20 (16.1)	124
Total	130 (80.2)	8 (4.9)	24 (14.8)	162
CRRT				
Level I	36 (94.7)	2 (5.3)	0 (0)	38
Level II	79 (63.7)	11 (8.9)	34 (27.4)	124
Total	115 (71.0)	13 (8.0)	34 (21.0)	162

The values are represented as *n* (%). HD—hemodialysis; CRRT—continuous renal replacement therapy.

## Data Availability

The Data That Support the Findings of this Study are Available from the National Emergency Medical Center of the Republic of Korea, but Restrictions Apply to the Availability of these Data, Which were Used under License for the Current Study and Thus are not Publicly Available.
